# Deep sequencing analysis of Human T cell lymphotropic virus type 1 (HTLV-1) long terminal repeat (LTR) region of tropical spastic paraparesis (TSP)/HTLV-1 associated myelopathy (HAM) and asymptomatic carriers

**DOI:** 10.1186/1742-4690-11-S1-P85

**Published:** 2014-01-07

**Authors:** Filipe FA Rego, Tulio de Oliveira, Bernardo Galvão-Castro, Luiz CJ Alcantara

**Affiliations:** 1 Gonçalo Moniz Research Center, Oswaldo Cruz Foundation, Salvador, Bahia, Brazil; 2 Africa Centre for Health and Population Studies, Nelson R Mandela School of Medicine, University of KwaZulu-Natal, Durban, KwaZulu-Natal, South Africa; 3 HTLV Center/ Bahia School of Medicine and Public Health/Bahia Foundation for Science Development, Salvador, Bahia, Brazil

## 

The aim of this study is to identify HTLV-1 minor strains that change the bind sites for transcriptional factors in the LTR promoter region. For this purpose 28 patients samples were analyzed, being 14 with TSP/HAM and 14 health carriers. The LTR fragments were submitted to the deep sequencing using the Ion Torrent methodology for a 200bp read in a 314 chip. We found 172 variations in these 28 patients samples. The single nucleotide polymorphisms (SNP) screening showed minor variants strains in the previously described transcriptional factor bind sites (TxRE-2 and 3, ETS binding sites and SP1 binding sites). In addition a SNP in the TATA box from an asymptomatic carrier sample was found in 90% of the sequences changing the site from TATAAA to TCTAAA (depth of 2632 nucleotides). Two asymptomatic carriers samples showed a 9 and 11 base pair deletion in all the sequences, being the 9 base pair deletion between the first and second TxRE and the 11 base pair deletion in the second TxRE. The number of minor variants was not constant (0 to 19) and was not related to proviral load or clinical status. An in silico analyze has being done in order to identify the motifs for transcriptional bind factor in those minor variants. The site directed mutagenesis followed by a luciferase assay will be performed to analyze the LTR activation in those strains.

